# The Prevalence of Epstein-Barr Virus in Plasma Cell Neoplasms is Higher in HIV-Positive Individuals

**DOI:** 10.1177/10668969221113490

**Published:** 2022-08-01

**Authors:** Ingrid H Penzhorn, Johann W Schneider, Candice Sher-Locketz

**Affiliations:** 1 Division of Anatomical Pathology, Department of Pathology, Faculty of Medicine and Health Sciences, National Health Laboratory Service, 98826 University of Stellenbosch, Tygerberg Hospital, Cape Town, South Africa; 2 Anatomical Pathology, 484973 PathCare, Cape Town, South Africa

**Keywords:** plasma cell neoplasm, plasmacytoma, Epstein-Barr virus, EBER-ISH, HIV, p24

## Abstract

Aims. Epstein-Barr virus (EBV) is causally associated with many hematolymphoid malignancies. This laboratory-based study aimed to establish the prevalence of EBV in plasma cell neoplasms in a large South African cohort and to determine whether there is any correlation between EBV-positivity and human immunodeficiency virus (HIV) status in patients with plasma cell neoplasms, including plasma cell myeloma and plasmacytoma (solitary plasmacytoma of bone and extraosseous plasmacytoma). Methods. This single-institution retrospective study included all patients with a histopathologic diagnosis of plasma cell neoplasm between 2003 and 2020. EBV-expression in the plasma cell neoplasms was assessed by EBV-encoded RNA (EBER) in situ hybridization (ISH) and correlated with HIV status. HIV status was determined by retrieving prior serologic results. Formalin-fixed paraffin-embedded tissue from HIV-unknown patients underwent HIV-1 p24 antibody testing. Results. Sixteen of 89 plasma cell neoplasms (18%) were EBV-positive. There was a significant correlation between EBV and HIV infection in plasma cell neoplasms, with 6/10 tumors from HIV positive patients showing EBV-positivity in tumor cells. The EBV-positive cohort was significantly younger than the EBV-negative group. Conclusion. EBV-positivity in plasma cell neoplasms in this study is higher than previously reported. The significant occurrence of EBV in plasma cell neoplasms from HIV-positive patients suggests a co-carcinogenic relationship between the two viruses.

## Introduction

Plasma cell myeloma and plasmacytoma are plasma cell neoplasms with similar histopathologic but different clinical characteristics. Plasmacytomas are usually solitary bone or extraosseous lesions with no or minimal (<10%) clonal plasma cells in the bone marrow, while plasma cell myeloma presents with multiple lytic bone lesions and invariable bone marrow involvement. Nearly all patients with plasma cell myeloma have monoclonal antibodies, or so-called M-protein, in urine or serum, with resultant end-organ damage (renal failure, anemia and hypercalcemia). M-protein is less commonly detected in plasmacytoma, and end-organ damage is absent by definition. The neoplastic cells encountered in these two entities typically display a plasmacytic phenotype resembling mature plasma cells. Although plasmacytic morphology is most commonly encountered, plasma cell neoplasms can also display anaplastic or plasmablastic morphology.^1^ The latter is defined by immature cells with a high nuclear/cytoplasm ratio, dispersed nuclear chromatin, prominent nucleoli and absent or inconspicuous perinuclear clearing.^2–4^

Epstein-Barr virus (EBV) is a ubiquitous gamma-herpesvirus that will infect more than 95% of humans during their lifetime.^5^ EBV is associated with many non-neoplastic and neoplastic entities. One of them is plasmablastic lymphoma—an aggressive B-cell lymphoma that can be a histologic mimicker of plasma cell neoplasms. Plasmablastic lymphoma and plasma cell neoplasms with plasmablastic morphology share similar cytologic features and plasmacytic immunoprofiles, complicating the histologic distinction between these entities.^6^ These overlapping features pose a diagnostic dilemma, as plasmablastic lymphoma and plasma cell neoplasms run diverging clinical courses and require different treatment protocols.^7–9^ Currently, the most reliable distinguishing factor is EBV-positivity in plasmablastic lymphoma, with The World Health Organization reporting EBV-encoded RNA (EBER) in situ hybridization (ISH) testing to be positive in 60–75% of plasmablastic lymphoma.^1^ The presence of EBV in plasma cell neoplasms is historically so unusual that single or low-number cases still warrant reporting in journals (Supplementary Table 1).

Competent T-cell immune surveillance is required to control EBV-infection and curb the production of “immortal” B lymphoblastoid cell lines that can lead to lymphoid malignancies.^5, 10^ Therefore, it is not surprising that immunodeficiency increases the risk of EBV-associated lymphoproliferative disorders.^11^ EBV-positive plasma cell neoplasms are usually reported in post-transplant patients^12–18^ and less commonly in HIV-positive patients,^3, 19–23^ with the prevalence dramatically lower in immunocompetent individuals.^20^ HIV predisposes infected individuals to many cancers, including several hematolymphoid malignancies. While plasmablastic lymphoma is considered an (EBV-driven) AIDS-defining malignancy, it is unclear if HIV-infection is causally associated with plasma cell neoplasms.^24–28^ Meta-analyses of studies reporting the incidence of plasma cell neoplasms in HIV-infected patients in high-income countries have revealed increased standardized incidence ratios^26, 27, 29^ with patients presenting at a younger age^24, 30^ and succumbing to a more aggressive disease course.^27^ Good quality epidemiological data on HIV and plasma cell neoplasms in low- and middle-income countries are much more challenging.^31^ Dhokotera et al^28^ did not report an increase in plasma cell myeloma in HIV-positive individuals after reviewing South African National Cancer Registry data for ten years.

This laboratory-based study aims to establish the prevalence of EBV in plasma cell neoplasms in a large South African cohort and to determine whether there is any correlation between EBV-positivity and HIV status in patients with plasma cell neoplasms.

## Materials and Methods

### Case Selection, Morphologic and Immunohistochemical Review

The study cohort included 97 patients diagnosed with plasmacytoma (solitary plasmacytoma of bone and extraosseous plasmacytoma) or plasma cell myeloma at a single South African institution between 1 January 2003 and 31 March 2020. Bone marrow aspirates and trephine biopsies were excluded. A pathologist and pathology trainee independently reviewed each tumor's histomorphology and immunohistochemical panels. The pathologist and trainee found no diagnostic discrepancies with the initial pathology reports. All EBV-positive tumors and all tumors included in the tissue microarrays were reviewed by a second pathologist. Tumors with a minor (less than 30%) component of blastic tumor cells were categorized as plasmacytic, while tumors comprising more than 30% of blastic cells were classified as plasmablastic.^9^ The minimum immunohistochemical requirements for inclusion of a tumor were positivity for multiple myeloma (MUM)-1 and CD138, evidence of immunoglobulin light chain restriction (kappa or lambda) and weak or absent staining for CD20. Bone marrow aspirate and serum/urine electrophoresis reports were reviewed as further support for diagnosing plasma cell myeloma (Supplemental material). Tumors with blastic morphology were only included if there was clinical consensus to support a diagnosis of plasmablastic myeloma; cases with reasonable concern for plasmablastic lymphoma were excluded. This study did not include reactive plasma cell lesions and other lymphoid neoplasms with plasmacytic differentiation, like extranodal marginal zone lymphoma. Cases with insufficient residual tumor tissue for performing EBER-ISH and/or p24 immunohistochemistry were excluded.

### Immune Status

HIV status was recorded based on available results of HIV enzyme-linked immunoassay (ELISA) testing or HIV viral load testing. Also included were tumors from patients with a known history of HIV infection and an available CD4 count. HIV status was recorded as positive, negative, or unknown. None of the patients had undergone solid organ or stem cell allograft transplants.

### Tissue Microarray Assembly

Formalin-fixed paraffin-embedded (FFPE) tissue blocks with sufficient tumor tissue were used to construct two tissue microarrays (TMA)s. The procedure entailed extracting 1mm diameter tumor tissue cores from suitable blocks using a UNITMA microarrayer (catalog #IW-UT06, immunohistochemistry (IHC) World, Ellicott City, MD, USA) and re-embedding these cores into a gridded paraffin block. Fifty tumors (one core per tumor) were successfully incorporated into the two TMAs. Ovarian, appendiceal and skin tissue were used as control place markers.

### EBV-Encoded Small RNA in Situ Hybridization

EBV-encoded small RNA (EBER) in situ hybridization (ISH) was performed on FFPE sections from tissue blocks (47 tumors) and sections from the TMAs (representing 50 tumors) using the Leica BOND Ready-to-use chromogenic EBER probe (Leica Biosystems, Newcastle upon Tyne, UK) and according to the supplier's protocol. An RNA control to demonstrate the presence of suitable RNA for hybridization was not used. Results were independently interpreted by three investigators using conventional light microscopy. Tumors were assigned as positive if more than 5% of neoplastic cells showed brown nuclear staining^32^ without confounding artefactual staining.

### Immunohistochemistry: p24 Antigen

HIV-1 p24 antibody recognizes part of a capsid gag protein unique to the human immunodeficiency virus.^33^ In this study, p24 immunohistochemistry was performed to determine whether HIV was present in tumoral tissue from patients in which the HIV status could not be conclusively determined from prior serologic testing or clinical information captured from laboratory request forms. Tumors from patients with an unknown HIV status were stained with anti-HIV-1 p24 antibody from Dako Diagnostics (Agilent Dako, Burlington, ON, Canada, 1:10) on the Dako Link Autostainer 48. Lymph node tissue from an HIV-positive patient was used as external control and showed positive cytoplasmic staining in follicular dendritic cells. Two tumors from HIV-positive patients were included in the TMAs as an “internal positive control” representing exclusive tumoral tissue.

### Statistical Analysis

Chi-squared, Fisher’s exact and Mann-Whitney U tests were performed using GraphPad Prism, version 5. Hypotheses were two-tailed, and *P*-values of <.05 were considered significant.

## Results

### Tumor Sites, Demographics, and Morphology

**Figure 1.**
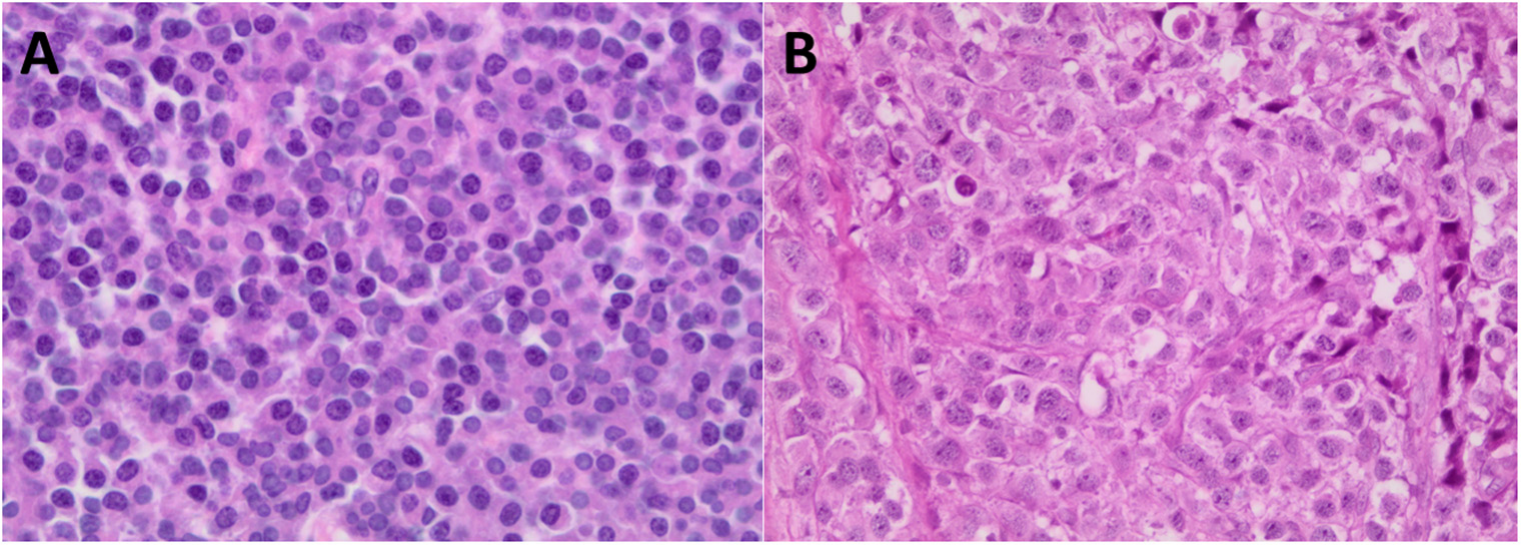
Plasma cell neoplasm histology, hematoxylin and eosin,  × 400. (A) Plasmacytic morphology. (B) Plasmablastic morphology.

The patient cohort comprised 54% men and 46% women (1.07:1) with a mean age of 55 years (Table 1). Of the 97 tumors included in the study, 64 biopsies were from bone sites and had undergone decalcification. The remainder of the biopsies were from the upper aerodigestive tract, lower respiratory tract, skin, liver, lymph nodes, submandibular salivary gland and soft tissue sites (Table 2). Plasmacytic morphology occurred in most tumors (Table 2; Figure 1A), with only 7/97 (7%) showing plasmablastic morphology (Table 2; Figure 1B).

**Table 1.** Patient Demographics and HIV Status.

Age^a^	Gender		HIV status	
11 to 84 (55)	Female 45 (46%)^b^	Male 52 (54%)^b^	Positive 10 (16%)^c^	Negative 56 (84%)^c^

^a^ Age range in years with mean; ^b^Total of 97 patients; ^c^Sixty-six patients had a known HIV status.

**Table 2.** Tumor Sites and Morphology.

Biopsy site	Axial skeleton	Appendicular skeleton	Bone, NOS	Liver	Upper aerodigestive tract	Lower respiratory tract	Soft tissue	Skin	Lymph node	Salivary gland
Plasmacytic^a^	29	29	1	2	9	2	13	2	2	1
Plasmablastic^b^	3	1	0	0	1	0	0	1	1	0

Number of cases with ^a^predominantly mature plasma cells (<30% plasmablastic cells); ^b^more than 30% plasmablastic cells.

### EBER-ISH

**Figure 2.**
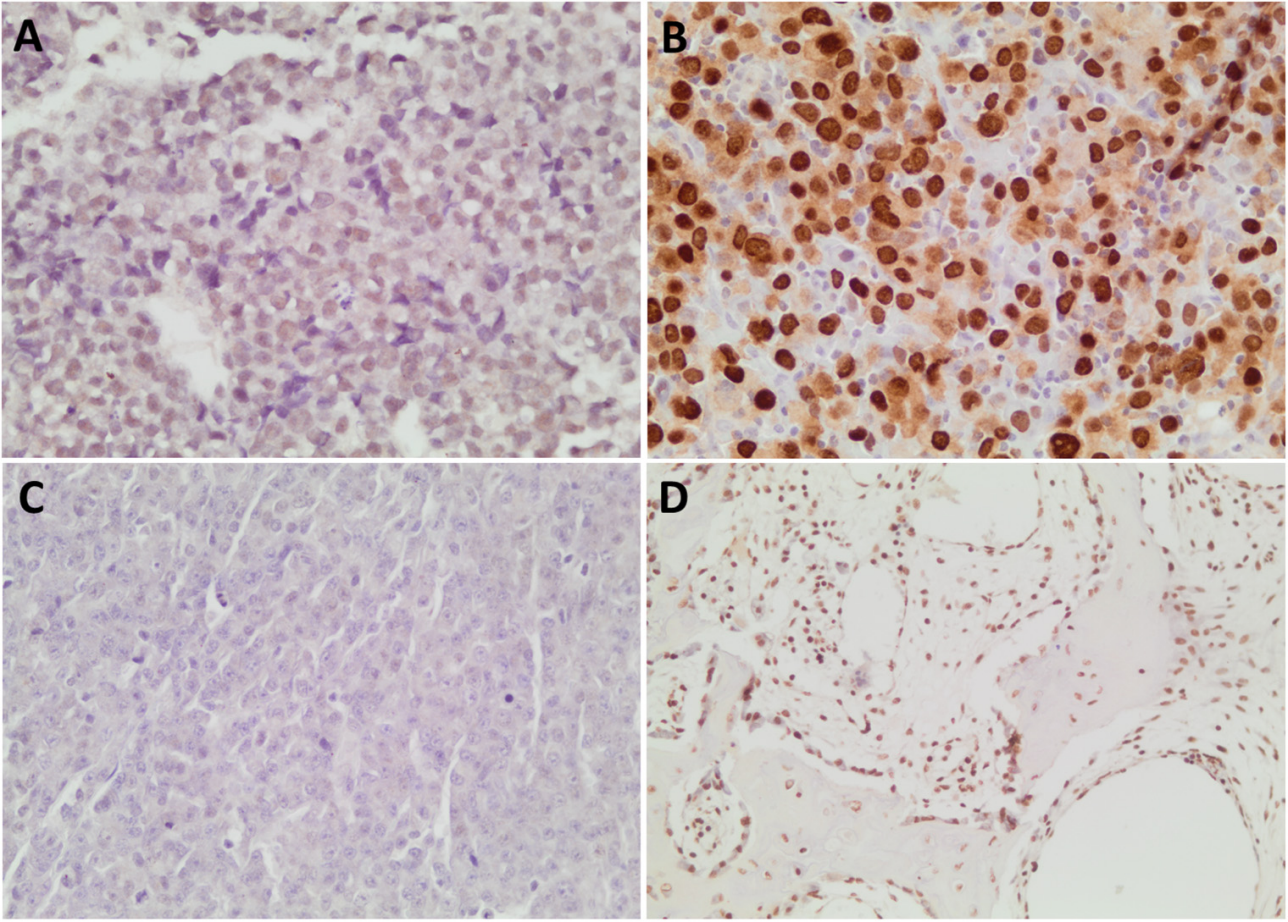
EBER-ISH stain. (A) Weak nuclear staining interpreted as equivocal, × 400. (B) Positive nuclear staining, × 400. (C) Negative nuclear staining, × 400. (D) Positive nuclear staining in stromal cells, endothelial cells, osteoblasts, and osteocytes, × 200.

Of the 97 tumors, eight were excluded from statistical analysis due to equivocal staining. The EBER-equivocal tumors showed staining artefacts which included nuclear staining mainly at the periphery of the tissue sections, weak nuclear staining (Figure 2A), stronger cytoplasmic than nuclear staining, and non-specific background staining confounding the interpretation of nuclear staining. Six of the eight tumors with equivocal staining had been decalcified. The external control tissue used for EBER-ISH testing was processed in the same laboratory and had not been decalcified; it showed crisp nuclear staining without background artefact. With the equivocal tumors removed, 16/89 tumors (18%) showed convincing nuclear staining (Table 3; Figure 2B), with the remainder having legitimate negative staining without excessive artefact (Figure 2C). There was no gender difference (*P*-value 1.0), but the EBV-positive cohort was significantly younger than the EBV-negative group (*P*-value .00124) (Table 3).

**Table 3.** EBV Status Versus Age and Gender.

Positive EBV-status^a^	EBV-status versus mean age in years*		EBV-status versus gender**			
	Negative	Positive	Negative		Positive	
16/89 (18%)	57.58	44.25	Male 38	Female 35	Male 8	Female 8

^a^ Based on EBER-ISH result; *P-value of Mann-Whitney U test is .00124. **P-value of chi-squared test is 1.0.

The EBV-positive tumors were significantly linked to positive HIV patient status (*P*-value .0027) (Table 4). There was no significant correlation between EBV status and plasmacytic versus plasmablastic morphology (*P*-value .106).

**Table 4.** EBV Versus HIV Status.

EBV-status in HIV-negative patients*		EBV-status in HIV-positive patients*		EBV-status in HIV-unknown patients^a^	
Negative	Positive	Negative	Positive	Negative	Positive
42	8	4	6	27	2

*P-value of chi-squared test is .0027; ^a^The two EBV-positive tumors with unknown HIV patient status were not included in the statistical analysis.

An interesting finding was that three tumors—two showing positive staining in tumor cells, one being negative—had convincing nuclear EBER-ISH staining in endothelial cells, stromal cells and even osteoblasts and osteocytes in the absence of any background staining (Figure 2D). All three of these tumors arose in bony sites (humerus, thoracic vertebra and iliac wing), and all three had undergone decalcification.

The three independent observers showed >90% correlation in their interpretation of the EBER-ISH stains.

### HIV Status and P24 Immunohistochemical Staining

Of the 97 plasma cell neoplasms included in this study, 64 (66%) had a known HIV patient status (Table 1). Ten patients were HIV-positive (16%), in line with the most recent estimate of a 14% HIV prevalence in South Africa.^34^ None of the tumors from patients with unknown HIV status that underwent HIV-1 p24 immunohistochemical testing showed positive staining. It is important to note that the two tumors with known HIV-positive patient status included in the TMAs also showed entirely negative p24 staining. Due to this finding of ‘false negative’ staining in the internal positive controls, the tumors with unknown patient status were not assigned a negative HIV status and instead remained HIV-unknown.

## Discussion

Our finding of 18% EBV-positivity in plasma cell neoplasms is higher than previously reported in larger-number studies. Chang et al^35^ had reported four of 58 plasma cell neoplasms to be EBV-positive (6.9%); Yan et al^36^ described 4/46 EBV-positive tumors (8.7%), and Nael et al^20^ reported 6/131 (4.6%). The historically uncommon association of EBV with plasma cell neoplasms, as opposed to some other B-cell lymphomas, is primarily ascribed to the absence of the EBV receptor CD21 on plasma cells.^12, 37, 38^ The tumorigenesis of plasma cell neoplasms is still incompletely understood, as myeloma cells are notoriously difficult to culture. Matsui et al^39^ have suggested that the ‘stem cells’ giving rise to plasma cell neoplasms are CD138-negative, CD20-positive B-cells that eventually differentiate into clonal mature CD138-positive, CD20-negative plasma cells. Whether plasma cell neoplasm “stem cells’ express CD21 receptors required for EBV infection has not yet been determined.^38^ The current incomplete understanding of the pathogenic role of EBV infection in plasma cell neoplasms is potentially hampering the effective treatment of this disease, as EBV-positive B-cell lymphomas are known to be biologically distinct from EBV-negative lymphomas, requiring different treatment approaches.^40^

Most EBV-related malignancies display latent EBV-infection, but the lytic phase is also implicated in oncogenesis.^41^ Four EBV-latency phases (0-III) have been described,^41, 42^ with their different gene expression and protein products implicated in varying aspects of oncogenesis.^43, 44^ EBERs are abundantly expressed during all four EBV latency phases,^45, 46^ and therefore, its presence does not distinguish between the different latency phases. EBER-positivity also does not confirm that EBV is indeed in the latent phase of infection. Although EBERs are generally thought to be downregulated (absent) during the lytic phase,^47^ Naidoo has reported co-expression of EBER with BamHI Z fragment leftward open reading frame (BZLF) 1, a lytic phase-specific gene, in diffuse large B-cell lymphoma.^48^ Latent membrane protein (LMP) 1 immunohistochemistry has only been performed on a handful of plasma cell neoplasm tumors (Supplementary Table 1). Therefore, further studies exploring other latency phase proteins (LMP2A/B, EBV-encoded nuclear antigens (EBNAs), non-transcribed BamHI-A rightward transcripts [BART] RNAs)^43^ and lytic phase gene expression in EBV-positive plasma cell neoplasms are required to characterize the nature of EBV-infection in plasma cell neoplasms.

The significant correlation of EBER-positivity with positive HIV status suggests a co-carcinogenic relationship between the two viruses. HIV does not primarily infect plasma cells,^31^ therefore other biologic mechanisms have been proposed to explain reports of increased incidences of plasma cell neoplasms in HIV-positive patients. These mechanisms include B-cell proliferation due to chronic antigenic stimulation by HIV proteins,^49^ the more potent effect of oncogenic viruses like EBV in impaired immunity and elevated levels of interleukin-6, which is an integral plasma cell growth factor associated with plasma cell neoplasm tumorigenesis.^30, 50–52^ As the vast majority of reports regarding EBV-positive plasma cell neoplasms originate in high-income countries (Supplementary Table 1), where HIV infection is a less common cause of immunosuppression than in low- and middle-income countries,^53^ data on the relationship between EBV and HIV in plasma cell neoplasms have been underrepresented in the literature. Better characterizing this relationship could potentially benefit the management of HIV patients, as plasma cell neoplasms in post-transplant immunosuppressed patients are known to behave more like post-transplant lymphoproliferative disorder (PTLD) B-cell lymphomas in prognosis and treatment response than plasma cell neoplasms encountered in immunocompetent patients.^16^ Whether this is also the case in HIV patients with plasma cell neoplasms remains to be seen.

Equivocal EBER-ISH staining was a limitation observed in eight tumors, of which six were decalcified bone specimens. Although RNA degradation due to decalcification could have negatively affected EBER-ISH testing, statistical analysis did not reveal a significant difference in equivocal staining between decalcified and non-decalcified tissue (*P*-value .71). Using control RNA probes to confirm RNA integrity after decalcification should be considered in future studies.

The finding of crisp nuclear EBER-ISH staining in stromal, endothelial and bone cells was unexpected. In vitro cell culture studies have shown EBV to be present in endothelial cells,^54^ and EBER-expression has been documented in endothelial cells in EBV-associated nasopharyngeal carcinoma.^45^ Only one in vivo study refers to EBER-ISH staining in tumoral stromal cells—this was reported in sclerosing angiomatoid nodular transformation (SANT) of the spleen. SANT is a non-neoplastic reactive lesion in which the stromal cells are considered part of the lesion;^55^ an entirely different biologic milieu than plasma cell neoplasms. The oncogenic qualities of EBV have been widely studied, focusing on its ability to evade the immune system through latency and its ability to create immortal B-cell lines. Less is known about its role in establishing or promoting a microenvironment where a neoplasm can flourish.^56^ Although the finding of stromal and endothelial staining is possibly non-specific, it could be worthwhile to explore in future studies.

Although some association between EBV-positivity and plasmablastic morphology in plasma cell neoplasms has been reported,^20, 35^ our data did not reveal a significant correlation.

P24 immunohistochemistry did not contribute to this study outcome. The most likely reason for the pervasive negative p24 staining, including the ‘false negative’ staining in tumors from patients with confirmed HIV-positive status, is the absence of cell types infected by HIV in plasma cell neoplasm tumoral tissue. HIV primarily infects CD4 + T-cells and dendritic cells,^57^ with B-cells and plasma cells seemingly spared.^31^ Germinal center follicular dendritic cells are the most reliable cells to express the p24 antigen in FFPE tissue derived from HIV-infected individuals.^58, 59^ Staining is also demonstrated in the mantle zone, intrafollicular and paracortical lymphocytes in lymphoid tissue. P24 staining has not been reported in epithelial cells, stromal cells and plasma cells.^58^ Another consideration for the absent p24 staining is p24’s specificity for the HIV-1 viral type. Although cross-reactivity between p24 and HIV-2 infected cells has been reported in cell culture studies,^60^ the DAKO p24 antibody is not expected to stain HIV-2 infected cells.^61^ This is unlikely to be a contributing factor, however, as HIV-1 infects most South African patients living with HIV.^62^

While the finding of 18% EBV-positivity in plasma cell neoplasms could potentially contribute to the reconsideration of pathologists’ reliance on EBER-ISH in distinguishing between plasma cell neoplasms with plasmablastic morphology and plasmablastic lymphoma, this study had several limitations. Due to its retrospective nature, clinical information, including radiology, the presence of end organ damage, detection of M-protein, and HIV status, could only be gleaned from laboratory records and the information provided to the pathologist at the time of biopsy. HIV status was unavailable in 34% of patients, and p24 immunohistochemistry did not contribute to determining HIV status in tumoral tissue. Also, the clinical outcome of EBV-positive versus EBV-negative patients, and HIV-positive versus HIV-negative patients, could not be determined. Another limitation was not using RNA probes in decalcified tissue to determine RNA integrity before performing EBER-ISH.

Several questions arose during the execution of this study, which might aid in guiding future research: In which latency phase is EBV when detected in plasma cell neoplasms? To what degree does decalcification affect the detection of EBER using situ hybridization? If EBV is genuinely present in stromal cells, does it play a role in establishing a microenvironment allowing for tumor development? Do HIV and EBV have a co-carcinogenic relationship, and how does that affect clinical outcomes?

## Supplemental Material

sj-docx-1-ijs-10.1177_10668969221113490 - Supplemental material for The Prevalence of Epstein-Barr Virus in Plasma Cell Neoplasms is Higher in HIV-Positive Individuals Click here for additional data file. Supplemental material, sj-docx-1-ijs-10.1177_10668969221113490 for The Prevalence of Epstein-Barr Virus in Plasma Cell Neoplasms is Higher in HIV-Positive Individuals by Ingrid H Penzhorn, Johann W Schneider and Candice Sher-Locketz in International Journal of Surgical Pathology
