# Supplementary material for: The Prevalence of Epstein-Barr Virus in Plasma Cell Neoplasms is Higher in HIV-Positive Individuals
Source: Int J Surg Pathol. 2022 Aug 1;31(5):564–71. doi: 10.1177/10668969221113490 (PMC10315871; doi:10.1177/10668969221113490)
Supplement: sj-docx-1-ijs-10.1177_10668969221113490 - Supplemental material for The Prevalence of Epstein-Barr Virus in Plasma Cell Neoplasms is Higher in HIV-Positive Individuals [file sj-docx-1-ijs-10.1177_10668969221113490.docx]

**Table 1 Reports of EBV-positive plasma cell myeloma and plasmactyoma 1989-2019**

| Ref.nr | Study year | Study site | Nr of cases | EBV+ | Immune status | EBER-ISH | Comments |
| --- | --- | --- | --- | --- | --- | --- | --- |
| 1 | 1989 | US | 1 | 1 | HIV | NP | Unspecified EBV DNA-ISH test |
| 2 | 1994 | US | 3 | 2 | HIV | NP | Unspecified EBV DNA-ISH test |
| 3 | 1994 | US | 1 | 1 | Transplant | Positive |  |
| 4 | 2000 | Spain | 1 | 1 | Transplant | Positive |  |
| 5 | 2005 | US | 1 | 1 | HIV | NP | EBV LMP1 was positive |
| 6 | 2006 | US | 1 | 1 | Transplant | Positive |  |
| 7 | 2007 | Taiwan | 58 | 16 | Competent | Positive |  |
| 8 | 2010 | US | 1 | 1 | Transplant | Uncertain | Unspecified EBV-ISH test |
| 9 | 2011 | Singapore | 1 | 1 | Competent | Positive |  |
| 10 | 2011 | Japan | 1 | 1 | Competent | Positive |  |
| 11 | 2012 | Mexico | 1 | 1 | Competent | Positive |  |
| 12 | 2012 | Japan | 1 | 1 | Competent | Positive |  |
| 13 | 2013 | US | 3 | 3 | 2 competent, 1 HIV | Positive | LMP1 positive in 1 case |
| 14 | 2013 | US | 18 | 7 | Transplant | Uncertain | Unspecified test |
| 15 | 2013 | US | 1 | 1 | HIV | Positive | LMP1 negative |
| 16 | 2013 | US | 9 | 7 | Transplant | Uncertain | EBER-ISH and/or LMP1 IHC were performed to assign status |
| 17 | 2014 | US | 1 | 1 | Competent | Positive |  |
| 18 | 2015 | US | 4 | 4 | Competent | Positive |  |
| 19 | 2015 | US | 1 | 1 | Transplant | Positive |  |
| 20 | 2017 | China | 46 | 4 | Competent | Positive |  |
| 21 | 2017 | France | 3 | 1 | Transplant | Positive |  |
| 22 | 2017 | US | 8 | 2 | 1 competent, 1 HIV | Positive |  |
| 23 | 2019 | US | 131 | 6 | 3 competent, 2 HIV, 1 transplant | Positive | Monoclonal gammopathy not included in this table. 4/6 positive cases also included in previous case reports^13,15^ |

NP = Not performed; ISH = In situ hybridisation; LMP = Latent membrane protein; PCR = Polymerase chain reaction; IHC = Immunohistochemistry

# **References for table 1**

1. Voelkerding KV, Sandhaus LM, Kim HC, et al. Plasma cell malignancy in the acquired immune deficiency syndrome. Association with Epstein-Barr virus. *Am J Clin Pathol*. 1989;92:222-8.

2. Kumar S, Kumar D, Schnadig VJ, et al. Plasma cell myeloma in patients who are HIV-positive. *Am J Clin Pathol*. 1994;102:633-9.

3. Joseph G, Barker RL, Yuan B, et al. Posttransplantation plasma cell dy1asias. *Cancer.* 1994;74:1959-64.

4. Ancín I, Sarrá J, Peris J, et al. Demonstration of Epstein-Barr virus in a case of multiple myeloma after renal transplantation. *Haematologica*. 2000;85:773-4.

5. Salarieh A, Rao C, Gottesman SR, et al. Plasma cell tumors in HIV-positive patients: Report of a case and review of the literature. *Leukemia & Lymphoma.* 2005;46:1067-1074.

6. Tcheng WY, Said J, Hall T, et al. Post-transplant multiple myeloma in a pediatric renal transplant patient. *Pediatr Blood Cancer*. 2006;47:218-23.

7. Chang ST, Liao YL, Lu CL, et al. Plasmablastic cytomorphologic features in plasma cell neoplasms in immunocompetent patients are significantly associated with EBV. *Am J Clin Pathol*. 2007;128:339-44.

8. Ninan MJ, Datta YH. Post-transplant lymphoproliferative disorder presenting as multiple myeloma. *Am J Hematol*. 2010;85:635-637.

9. Yan B, Tan SY, Yau EX, et al. EBV-positive plasmacytoma of the submandibular gland--report of a rare case with molecular genetic characterization. *Head Neck Pathol*. 2011;5:389-394.

10. Sasaki S, Hashimoto K, Nakatsuka S, et al. Plasmablastic extramedullary plasmacytoma associated with Epstein-Barr virus arising in an immunocompetent patient with multiple myeloma. *Intern Med*. 2011;50:2615-2620.

11. Garcia-Alvarez KG, Garibaldi-Covarrubias R, Flores-Marquez MR, et al. Plasma cell myeloma associated with Epstein-Barr virus infection in an 11-year-old girl. *Pediatr Dev Pathol*. 2012;15:339-342.

12. Saito M, Morioka M, Izumiyama K, et al. Epstein-Barr virus-positive ileal extraosseous plasmacytoma containing plasmablastic lymphoma components with CD20-positive lymph node involvement. *Int J Gen Med*. 2012;5:715.

13. Pasch W, Wu W, Bach D, et al. Epstein–Barr virus expression in plasma cell neoplasms and its association with plasmablastic morphologic features. *J Hematopathol*. 2013;6:213-218.

14. Engels EA, Clarke CA, Pfeiffer RM, et al. Plasma cell neoplasms in US solid organ transplant recipients. *Am J Transplant*. 2013;13:1523-1532.

15. Wu W, Pasch W, Zhao X, et al. Extraosseous plasmacytoma with an aggressive course occurring solely in the CNS. *Neuropathology*. 2013;33:320-3.

16. Karuturi M, Shah N, Frank D, et al. Plasmacytic post-transplant lymphoproliferative disorder: A case series of nine patients. *Transpl Int*. 2013;26:616-622.

17. Scarberry K, Jegalian A, Valent J, et al. Solitary extramedullary plasmacytoma of the penis. *Urol Ann*. 2014;6:242-243.

18. Loghavi S, Khoury JD, Medeiros LJ. Epstein-Barr virus-positive plasmacytoma in immunocompetent patients. *Histopathology*. 2015;67:225-234.

19. Wilberger AC, Prayson RA. Intracranial involvement of posttransplant lymphoproliferative disorder multiple myeloma. *J Clin Neurosci*. 2015;22:1850-1851.

20. Yan B, Tan SY, Yau EX, et al. EBV-positive plasmacytoma of the submandibular gland-report of a rare case with molecular genetic characterization. *Head Neck Pathol*. 2011;5:389-394.

21. Kormann R, Francois H, Moles T, et al. Plasma cell neoplasia after kidney transplantation: French cohort series and review of the literature. *PLoS ONE*. 2017;12:e0179406.

22. Marks E, Shi Y, Wang Y. CD117 (KIT) is a useful marker in the diagnosis of plasmablastic plasma cell myeloma. *Histopathology*. 2017;71:81-88.

23. Nael A, Wu WW, Siddiqi I, et al. Epstein-Barr virus association with plasma cell neoplasms. *Histol Histopathol*. 2019;34:655-662.

**Raw data**

| **Study Nr** | **Sex** | **Age** | **HIV** | **EBER** | **Site** | **Morphology** | **M-protein or BM**** |
| --- | --- | --- | --- | --- | --- | --- | --- |
| IPP001 | F | 57 | Negative | Positive | Clavicle | plasmacytic | Present |
| IPP002 | M | 49 | Unknown | Negative | T9 vertebra | plasmacytic | Present |
| IPP004 | F | 74 | Negative | Positive | Distal humerus | plasmacytic | Negative or insufficient information |
| IPP005 | M | 56 | Positive | Negative | Right lung | plasmacytic | Present |
| IPP006 | F | 84 | Unknown | Negative | T12 vertebra | plasmacytic | Negative or insufficient information |
| IPP007 | M | 64 | Unknown | Negative | Nasal polyp | plasmacytic | Negative or insufficient information |
| IPP008 | F | 46 | Unknown | Negative | Left femur | plasmacytic | Present |
| IPP009 | F | 48 | Negative | Negative | Left humerus | plasmacytic | Negative or insufficient information |
| IPP011 | M | 57 | Negative | Negative | Right humerus | plasmacytic | Negative or insufficient information |
| IPP012 | F | 60 | Negative | Negative | Sacrum | plasmacytic | Negative or insufficient information |
| IPP013 | F | 49 | Negative | Negative | Ant. chest wall | plasmacytic | Negative or insufficient information |
| IPP014 | M | 60 | Unknown | Negative | Pelvis | Plasmacytic | Negative or insufficient information |
| IPP015 | M | 33 | Positive | Positive | C3 vertebra | plasmacytic | Negative or insufficient information |
| IPP017 | F | 48 | Negative | Negative | T3 vertebra | plasmablastic* | Present |
| IPP018 | F | 73 | Unknown | Negative | Ant. chest wall | plasmacytic | Present |
| IPP019 | M | 46 | Negative | Positive | T6 vertebra | plasmacytic | Present |
| IPP020 | F | 55 | Negative | Positive | Rib/vertebra | plasmacytic | Present |
| IPP021 | F | 49 | Positive | Positive | Anterior chest wall | plasmacytic | Present |
| IPP022 | M | 60 | Negative | Negative | Sacrum | plasmablastic* | Present |
| IPP023 | M | 50 | Negative | Positive | Right clavicle | plasmacytic | Negative or insufficient information |
| IPP024 | F | 59 | Unknown | Negative | Right humerus | plasmacytic | Negative or insufficient information |
| IPP025 | F | 52 | Negative | Negative | Sacrum | plasmacytic | Negative or insufficient information |
| IPP026 | M | 48 | Positive | Negative | Nasal polyp | plasmacytic | Negative or insufficient information |
| IPP027 | F | 68 | Unknown | Negative | Left femur | plasmacytic | Present |
| IPP028 | M | 77 | Unknown | Negative | L1 vertebra | plasmacytic | Negative or insufficient information |
| IPP030 | M | 75 | Unknown | Negative | Neck, soft tissue | plasmacytic | Negative or insufficient information |
| IPP031 | M | 53 | Negative | Negative | T1 vertebra | plasmacytic | Present |
| IPP032 | F | 78 | Unknown | Negative | Left humerus | plasmacytic | Present |
| IPP033 | M | 74 | Negative | Equivocal | Right humerus | plasmacytic | Present |
| **Study Nr** | **Sex** | **Age** | **HIV** | **EBER** | **Site** | **Morphology** | **M-protein or BM**** |
| IPP034 | M | 23 | Negative | Negative | L4, paraspinal | plasmacytic | Negative or insufficient information |
| IPP035 | F | 57 | Negative | Negative | Mandible | plasmacytic | Present |
| IPP036 | F | 63 | Unknown | Negative | Right humerus | plasmacytic | Present |
| IPP037 | M | 59 | Negative | Negative | Sacrum | plasmacytic | Present |
| IPP038 | M | 64 | Unknown | Negative | Right humerus | plasmacytic | Negative or insufficient information |
| IPP039 | M | 53 | Unknown | Negative | Thoracic vertebra | plasmacytic | Negative or insufficient information |
| IPP040 | M | 59 | Unknown | Negative | Subment. lymph node | plasmacytic | Negative or insufficient information |
| IPP043 | M | 52 | Negative | Equivocal | Scapula | plasmacytic | Negative or insufficient information |
| IPP045 | M | 43 | Unknown | Equivocal | Left humerus | plasmacytic | Negative or insufficient information |
| IPP046 | F | 55 | Unknown | Negative | C7 vertebra | plasmacytic | Negative or insufficient information |
| IPP047 | F | 61 | Negative | Positive | Right femur | plasmacytic | Present |
| IPP048 | F | 57 | Negative | Negative | Anterior chest wall | plasmacytic | Negative or insufficient information |
| IPP049 | M | 43 | Positive | Positive | T9 vertebra | plasmacytic | Negative or insufficient information |
| IPP050 | M | 65 | Negative | Negative | Nasopharynx | plasmacytic | Negative or insufficient information |
| IPP051 | F | 63 | Unknown | Negative | Right humerus | plasmacytic | Present |
| IPP052 | F | 40 | Negative | Negative | T10 vertebra | plasmacytic | Negative or insufficient information |
| IPP053 | F | 34 | Negative | Negative | L1, paraspinal mass | plasmacytic | Present |
| IPP054 | M | 80 | Unknown | Equivocal | Anterior chest wall | plasmacytic | Negative or insufficient information |
| IPP055 | M | 36 | Negative | Equivocal | LIVER | Plasmacytic | Present |
| IPP056 | M | 37 | Negative | Negative | Submand. saliv. gland | plasmacytic | Negative or insufficient information |
| IPP057 | F | 60 | Positive | Negative | Right hip | plasmacytic | Present |
| IPP058 | M | 13 | Negative | Positive | Nasal polyp | plasmablastic* | Negative or insufficient information |
| IPP059 | M | 67 | Unknown | Negative | Iliac wing | plasmacytic | Present |
| IPP060 | F | 57 | Unknown | Positive | Iliac wing | plasmacytic | Present |
| IPP061 | F | 65 | Unknown | Negative | Skull/brain | plasmacytic | Negative or insufficient information |
| IPP062 | F | 61 | Unknown | Equivocal | T7 Vertebra | plasmacytic | Present |
| IPP063 | M | 55 | Unknown | Negative | Thoracic vertebra | plasmacytic | Negative or insufficient information |
| IPP064 | M | 45 | Negative | Equivocal | T5 vertebra | plasmacytic | Negative or insufficient information |
| IPP065 | M | 79 | Negative | Negative | Cervical lymph node | plasmacytic | Present |
| **Study Nr** | **Sex** | **Age** | **HIV** | **EBER** | **Site** | **Morphology** | **M-protein or BM**** |
| IPP066 | F | 11 | Unknown | Positive | Trachea/bronchus | plasmablastic* | Negative or insufficient information*** |
| IPP067 | F | 62 | Negative | Negative | Scalp, eroding skull | plasmacytic | Present |
| IPP068 | M | 55 | Negative | Negative | Anterior chest wall | plasmacytic | Present |
| IPP069 | M | 68 | Negative | Negative | mucosal | plasmacytic | Negative or insufficient information |
| IPP073 | M | 37 | Negative | Negative | Radius/ulna | plasmacytic | Negative or insufficient information |
| IPP074 | M | 48 | Positive | Positive | Right femur | plasmablastic* | Present |
| IPP075 | M | 46 | Positive | Positive | Left femur | plasmacytic | Present |
| IPP077 | F | 53 | Negative | Negative | Bone | plasmacytic | Present |
| IPP078 | M | 60 | Negative | Negative | Sternal mass | plasmacytic | Present |
| IPP079 | M | 29 | Negative | Negative | Paraspinal area | plasmacytic | Present |
| IPP080 | M | 44 | Negative | Negative | Sacrum | plasmablastic* | Present |
| IPP081 | M | 82 | Negative | Negative | Paraspinal area | plasmacytic | Negative or insufficient information |
| IPP082 | F | 65 | Negative | Negative | T8 vertebra | plasmacytic | Negative or insufficient information |
| IPP083 | M | 48 | Negative | Negative | Liver | plasmacytic | Present |
| IPP084 | M | 65 | Negative | Negative | T10 vertebra | plasmacytic | Negative or insufficient information |
| IPP085 | F | 48 | Negative | Negative | Right femur | plasmacytic | Present |
| IPP086 | F | 58 | Unknown | Negative | Nasopharyngeal | plasmacytic | Negative or insufficient information |
| IPP087 | F | 70 | Unknown | Negative | T10 vertebra | plasmacytic | Negative or insufficient information |
| IPP088 | F | 62 | Negative | Negative | C2 vertebra | plasmacytic | Negative or insufficient information |
| IPP089 | M | 53 | Unknown | Negative | Left orbit | plasmacytic | Negative or insufficient information |
| IPP090 | F | 59 | Negative | Negative | Skin | plasmacytic | Present |
| IPP091 | M | 19 | Negative | Negative | Right pelvis | plasmacytic | Present |
| IPP092 | F | 65 | Negative | Negative | Sphenoidal sinus | plasmacytic | Present |
| IPP093 | M | 67 | Unknown | Negative | Left femur | plasmacytic | Present |
| IPP094 | M | 73 | Unknown | Negative | Nasal polyp | plasmacytic | Present |
| IPP095 | M | 62 | Negative | Negative | Right femur | plasmacytic | Present |
| IPP096 | M | 65 | Negative | Negative | Nasal polyp | plasmacytic | Present |
| IPP097 | M | 39 | Positive | Negative | T9 vertebra | plasmacytic | Negative or insufficient information |
| IPP098 | F | 79 | Negative | Negative | Oral cavity | plasmacytic | Negative or insufficient information |
| IPP099 | M | 52 | Unknown | Negative | Right shoulder, soft tissue | plasmacytic | Negative or insufficient information |
| IPP0100 | F | 53 | Unknown | Equivocal | Left femur | plasmacytic | Present |
| IPP0101 | F | 55 | Negative | Negative | Right ilium | plasmacytic | Present |
| **Study Nr** | **Sex** | **Age** | **HIV** | **EBER** | **Site** | **Morphology** | **M-protein or BM**** |
| IPP0102 | M | 61 | Negative | Negative | Left hip soft tissue | plasmacytic | Present |
| IPP0104 | M | 37 | Negative | Positive | Left hip soft tissue | plasmacytic | Present |
| IPP0105 | F | 53 | Negative | Negative | Right tibia | plasmacytic | Present |
| IPP0106 | F | 28 | Positive | Positive | Anterior chest wall | plasmacytic | Present |
| IPP0107 | F | 58 | Negative | Negative | Orbit | plasmacytic | Present |
| IPP0108 | F | 58 | Negative | Negative | Skin, right flank | plasmablastic* | Present |
| IPP0109 | F | 60 | Unknown | Negative | Right femur | plasmacytic | Present |

*Plasmablastic tumors were only included if they had sufficient clinical information to exclude a diagnosis of plasmablastic lymphoma. E.g., History of multiple lytic bone lesions and renal failure, or history given as “known with multiple myeloma”, together with the presence of M-protein and/or bone marrow involvement.

**Presence of monoclonal antibodies in either serum or urine and/or bone marrow (BM) involvement by clonal plasma cells

***This was the only case with blastic morphology, without M-protein or bone marrow involvement to support a diagnosis of plasma cell myeloma. This case was referred to international colleagues at Harvard and the clinicopathological consensus was that it represented a solitary plasmacytoma with blastic morphology, rather than a plasmablastic lymphoma.
